# Clinical Implication of HIF-PH Inhibitor in Patients with Heart Failure, Chronic Kidney Disease, and Renal Anemia

**DOI:** 10.3390/jcm13247619

**Published:** 2024-12-13

**Authors:** Yuki Hida, Teruhiko Imamura, Koichiro Kinugawa

**Affiliations:** Second Department of Internal Medicine, University of Toyama, Toyama 930-8555, Japan

**Keywords:** renal anemia, hemodynamics, chronic kidney disease, cardio-renal syndrome

## Abstract

**Background:** Hypoxia-inducible factor-prolyl hydroxylase (HIF-PH) inhibitors have been developed as a treatment for renal anemia. However, their therapeutic impact on patients with concomitant heart failure remains uncertain. We investigated the impact of HIF-PH inhibitors on improving renal anemia and associated clinical outcomes in patients with heart failure. **Methods:** Patients with both heart failure and renal anemia who received HIF-PH inhibitors were retrospectively analyzed over a six-month follow-up period. Hemoglobin levels and other clinical parameters were compared between the six-month pre-treatment period without HIF-PH inhibitors and the six-month treatment period with HIF-PH inhibitors. **Results:** A total of 69 patients (median age 82 years, 27 male) were included. Baseline hemoglobin was 9.2 (8.8, 10.3) g/dL, baseline plasma B-type natriuretic peptide level was 264 (156, 372) pg/mL, and baseline estimated glomerular filtration rate was 29.1 (19.0, 35.1) mL/min/1.73 m^2^. Hemoglobin levels declined during the pre-treatment period from 10.5 (9.4, 11.5) g/dL to 9.2 (8.8, 10.3) g/dL (*p* < 0.001) but subsequently increased to 10.9 (10.1, 12.0) g/dL following six months of HIF-PH inhibitor treatment (*p* < 0.001). This increase in hemoglobin was accompanied by a reduction in plasma BNP levels, improved renal function, and reduced systemic inflammation (*p* < 0.05 for all). **Conclusions:** HIF-PH inhibitors demonstrated efficacy in this cohort of patients with heart failure, with associated improvements in heart failure severity, renal function, and systemic inflammation.

## 1. Introduction

Despite advances in guideline-directed medical therapy, morbidity and mortality in patients with heart failure remain unacceptably high [1]. A critical approach to improving clinical outcomes may lie in addressing comorbidities such as anemia [2].

Anemia is highly prevalent in patients with chronic kidney disease (CKD), primarily due to diminished erythropoietin synthesis [3]. In non-dialysis CKD patients, severe anemia has been linked to accelerated progression to end-stage kidney disease. Approximately 60% of patients with heart failure experience comorbid anemia [4], driven by a range of factors including renal anemia progression, iron utilization deficiency, impaired iron absorption, systemic congestion-related hemodilution, systemic inflammation, and bone marrow dysfunction [5]. Notably, the presence of heart failure-related inflammation facilitates erythropoietin resistance and iron metabolism dysfunction. Conversely, anemia impairs organ oxygenation and increases cardiac workload, thereby progressing the heart failure burden [5]. Needless to say, heart failure patients often complicate CKD due to reduced organ perfusion, renal congestion, and similar risk factors. The impaired renal function is associated with volume overload, particularly on the heart. The presence of CKD is an independent risk factor for mortality and morbidity in patients with heart failure.

These interconnected mechanisms create a self-perpetuating cycle of heart failure, CKD, and anemia, termed the cardio-renal anemia syndrome [6]. Breaking this cycle is essential for effective heart failure management, with anemia treatment being a promising intervention point [7].

Erythropoiesis-stimulating agents (ESAs) are widely used to treat renal anemia, with efficacy demonstrated across various patient populations [8]. However, a standardized approach to treating renal anemia in heart failure patients has not been established, particularly in light of adverse clinical outcomes associated with ESA therapy in this population. For example, the RED-HF trial, which examined ESA effects in patients with anemia and heart failure, found no benefit of ESA over placebo in reducing cardiovascular mortality and reported, rather, an increased risk of thromboembolism with ESA use [9].

As a result, the American College of Cardiology Foundation/American Heart Association guidelines provide a Class III recommendation against ESA use in heart failure patients with anemia [10], and the European Society of Cardiology advises against ESA use in this context [11]. Similarly, the Japanese Circulation Society guidelines advise against ESA therapy for treating anemia in heart failure patients with a Class III [12]. Consequently, therapeutic approaches for renal anemia in heart failure remain undefined so far.

Hypoxia-inducible factor-prolyl hydroxylase (HIF-PH) inhibitors represent a novel therapeutic class for renal anemia that has a completely different pathophysiological impact from conventional ESA [13]. These agents inhibit prolyl hydroxylase activity, stabilizing HIF-alpha and promoting endogenous erythropoietin production, thereby enhancing hemoglobin and erythrocyte synthesis. Recent randomized controlled trials have shown that HIF-PH inhibitors are non-inferior to ESAs in cardiovascular safety and hemoglobin improvement [14,15,16].

However, few studies have assessed the feasibility and efficacy of HIF-PH inhibitors in treating renal anemia specifically in heart failure patients [17,18]. No guidelines mention the benefit/non-benefit of HIF-PH inhibitors in the heart failure cohort. The unique mechanism of HIF-PH inhibitors, which increases endogenous erythropoietin and addresses iron utilization dysfunction, may offer particular benefits for this population, given that these factors are primary contributors to anemia in heart failure [19]. Conversely, systemic inflammation—a common feature in heart failure—may reduce erythropoietin production and impair bone marrow erythropoiesis, potentially limiting the efficacy of HIF-PH inhibitors. Furthermore, overexpression of HIF-1 alpha and HIF-2 alpha following the administration of HIF-PH inhibitors may result in cardiomyopathy development [20].

In this study, we investigated the feasibility and efficacy of HIF-PH inhibitors for managing renal anemia in heart failure patients by comparing clinical parameters during the treatment period with those observed in the pre-treatment period (i.e., an intra-group comparison).

## 2. Methods

### 2.1. Patient Selection

This retrospective study included patients with heart failure, CKD, and renal anemia who received HIF-PH inhibitor therapy for renal anemia and were followed for at least six months. Heart failure was diagnosed according to the Framingham criteria in patients receiving guideline-directed medical therapy as tolerated [21]. Renal anemia was defined as a hemoglobin level of 11 g/dL or below, CKD stage 3b or higher, negative fecal occult blood test results, and anemia not attributed to other specific causes including iron deficiency, pernicious anemia, hemolysis, vitamin B12 deficiency, and folic acid deficiency [22].

Patients were excluded if they had undergone urgent percutaneous or surgical interventions within the previous six months, were dependent on hemodialysis, or experienced significant bleeding requiring transfusion during the observation period. Patients with a history of malignancy or active malignancy were also excluded, as they did not receive HIF-PH inhibitors. Patients who had previously received ESAs were excluded as well. Written informed consent was obtained from all participants, and the study protocol was approved by the Institutional Ethics Committee (R2015154, 11 April 2016). Data were retrospectively retrieved from the institutional medical chart and the data set for the present study was constructed.

### 2.2. Study Design

Clinical data were collected at three time points: six months prior to treatment, baseline, and six months after the initiation of HIF-PH inhibitor therapy, creating two observation periods (pre-treatment and on-treatment). The primary outcome was the trend in hemoglobin levels, with secondary outcomes including trends in other clinical parameters, as detailed below.

### 2.3. HIF-PH Inhibitor Treatment

HIF-PH inhibitors were initiated at baseline for the treatment of renal anemia. The choice to commence HIF-PH inhibitor therapy was at the discretion of the attending physicians. Four clinically available HIF-PH inhibitors were used as per physician discretion: daprodustat (Kyowa Kirin Co., Ltd., Tokyo, Japan), vadadustat (Mitsubishi Tanabe Pharma Co., Osaka, Japan), roxadustat (Astellas Pharma Inc., Tokyo, Japan), and molidustat (Bayer Yakuhin, Ltd., Osaka, Japan). Initial dosing followed manufacturer recommendations (daprodustat: 4 mg/day; vadadustat: 300 mg/day; roxadustat: 70 mg/day, three times weekly; molidustat: 25 mg/day) and was adjusted to maintain hemoglobin levels between 10 and 12 g/dL. Oral iron supplementation was initiated for patients with iron deficiency, defined as a serum ferritin level <100 ng/mL or transferrin saturation <20%.

### 2.4. Data Collection

Demographic data, comorbidities, laboratory findings, echocardiographic results, and medication information were collected at baseline, just before HIF-PH inhibitor initiation. Laboratory and medication data were also gathered six months before and six months after HIF-PH inhibitor initiation. Patients were followed at our outpatient clinic or affiliated institutes by board-certified cardiologists at scheduled intervals.

### 2.5. Statistical Analysis

All statistical analyses were conducted using SPSS Statistics 26 (IBM, Armonk, NY, USA), with a two-sided significance level of *p* < 0.05 considered statistically significant. Continuous variables were presented as median and interquartile range (IQR), while categorical variables were expressed as frequencies and percentages.

For the primary outcome, hemoglobin trends over the pre-treatment and treatment periods were assessed using the Friedman test, with post hoc Wilcoxon signed-rank tests used for pairwise comparisons. Changes in hemoglobin levels between the pre-treatment and on-treatment periods were compared using the Mann–Whitney U test.

Other continuous variables were similarly compared across time points using the Friedman test and post hoc Wilcoxon signed-rank tests. For categorical variables, the Cochran Q test was applied for trend analysis, and, if significant, the McNemar test was used for pairwise comparisons.

Linear regression analysis was conducted to identify baseline variables associated with changes in hemoglobin levels during the six-month HIF-PH inhibitor therapy. Variables significant in univariable analyses were included in a multivariable analysis using a forced entry method.

## 3. Results

### 3.1. Baseline Characteristics

A total of 69 patients were included in the study. Baseline characteristics prior to initiating HIF-PH inhibitor therapy are summarized in Table 1. The types of HIF-PH inhibitors used were as follows: 39 patients received daprodustat, 20 vadadustat, 7 roxadustat, and 3 molidustat. No patients experienced adverse events, including thromboembolic events or death, during the treatment period. All patients completed six months of HIF-PH inhibitor therapy, except for two who discontinued: one on day 148 due to transfer to another facility and the other on day 168 due to anemia improvement.

The median age was 82 years (IQR: 78–85) and 27 patients (39%) were male. The plasma B-type natriuretic peptide level was 264 pg/mL (IQR: 156–372) and the estimated glomerular filtration rate was 29.1 mL/min/1.73 m² (IQR: 19.0–35.1). The serum C-reactive protein level was 0.31 mg/dL (IQR: 0.03–1.20) and the hemoglobin level was 9.2 g/dL (IQR: 8.8–10.3). A subset of patients (22%) had a left ventricular ejection fraction of <40%, with the remainder having mildly reduced or preserved ejection fraction. Patients received guideline-directed medical therapy as tolerated based on their vital signs, electrolyte levels, and renal function. Three-fourths received furosemide, and half received tolvaptan. No patients received intravenous inotropes, and none were classified as New York Heart Association functional class IV.

### 3.2. Hemoglobin Level Trajectory

**Figure 1:**
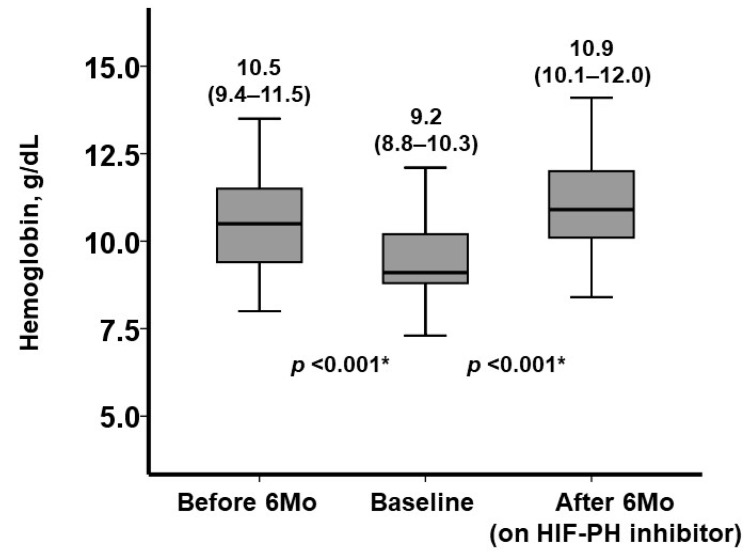
Trajectory of hemoglobin levels at three time points: six months before the initiation of HIF-PH inhibitors, baseline just before the initiation of HIF-PH inhibitors, and six months after the initiation of HIF-PH inhibitors (on treatment). When the trend analysis using Friedman test reached statistical significance, post hoc Wilcoxon signed-rank test was performed for two different time point data. * *p* < 0.05.

**Figure 2:**
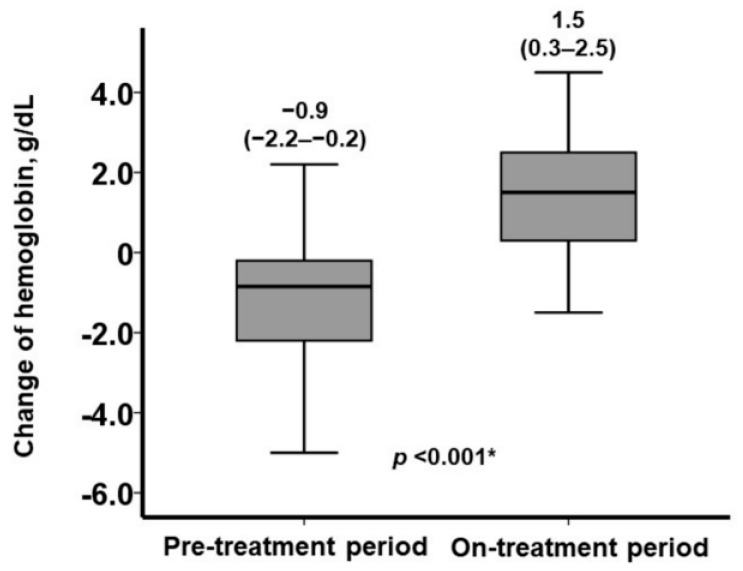
Changes in hemoglobin levels during six-month pre-treatment period and six-month on-treatment period. * *p* < 0.05 by Mann–Whitney U test.

During the six-month pre-treatment period without HIF-PH inhibitors, hemoglobin levels declined significantly from 10.5 g/dL (IQR: 9.4–11.5) to 9.2 g/dL (IQR: 8.8–10.3, *p* < 0.001; Figure 1). Following the initiation of HIF-PH inhibitor treatment, their doses were adjusted according to the hemoglobin levels. Six months later, the final dose of HIF-PH inhibitors was increased in most participants (91%). As a result, hemoglobin levels significantly increased to 10.9 g/dL (IQR: 10.1–12.0, *p* < 0.001). The change in hemoglobin level was significantly greater during the treatment period compared to the pre-treatment period: 1.5 g/dL (IQR: 0.3–2.5) versus −0.9 g/dL (IQR: −2.2–−0.2, *p* < 0.001; Figure 2).

Of the patients, 39 received daprodustat, 20 received vadadustat, and the remainder received other types of HIF-PH inhibitors. The hemoglobin increase was significantly higher in patients receiving daprodustat than in those receiving vadadustat: 1.9 g/dL (IQR: 1.1–2.9) versus 0.4 g/dL (IQR: 0.0–1.6, *p* = 0.003). The hemoglobin increases of other patients receiving roxadustat (N = 7) and molidustat (N = 3) were 2.0 (1.4, 2.1) g/dL and 1.4 (0.6, 1.5) g/dL, respectively.

### 3.3. Other Clinical Parameters

The eGFR declined significantly during the pre-treatment period and subsequently increased following six months of HIF-PH inhibitor therapy (*p* < 0.001 for both; Table 2). Plasma BNP levels remained stable during the pre-treatment period (*p* = 0.43) but significantly decreased during the treatment period (*p* = 0.018). Serum C-reactive protein levels were unchanged during the pre-treatment period (*p* = 0.82) but showed a significant reduction during the treatment period (*p* < 0.001).

Most echocardiographic parameters remained unchanged during both the pre-treatment and treatment periods, except for the prevalence of moderate or greater tricuspid regurgitation. The prevalence of tricuspid regurgitation increased significantly during the pre-treatment period (*p* = 0.021) and decreased during the treatment period (*p* = 0.008; Table 3).

The prescription rates for major heart failure medications remained unchanged throughout both the pre-treatment and treatment periods (*p* > 0.05 for all; Table 4). Diuretic doses also remained stable across both observation periods (*p* > 0.05 for all).

### 3.4. Variables Associated with Hemoglobin Increase During HIF-PH Inhibitor Therapy

During the six-month HIF-PH inhibitor therapy, the median increase in hemoglobin level was 1.5 g/dL (IQR: 0.3–2.5). Among nine potential variables, lower baseline hemoglobin level and lower E/e’ ratio were independently associated with the increase in hemoglobin levels (*p* < 0.001 and *p* = 0.002, respectively; Table 5).

## 4. Discussion

This study evaluated the effects of HIF-PH inhibitors on hemoglobin levels and other clinical parameters in patients with renal anemia and heart failure over a six-month treatment period. The results showed significant increases in hemoglobin levels, reductions in plasma B-type natriuretic peptide and C-reactive protein levels, and improved estimated glomerular filtration rate following HIF-PH inhibitor treatment, without any observed drug-related adverse events.

### 4.1. Rationale for Study Design

Previous studies on HIF-PH inhibitors for renal anemia in heart failure patients were limited in duration and scope without control arms [16,17]. Given ethical challenges in conducting placebo-controlled trials, this study utilized a six-month pre-treatment period as a self-control arm, instead of prospectively preparing a control arm without HIF-PH inhibitors (it is ethically challenging to manage renal anemia without HIF-PH inhibitors in the current era). This design allowed for comparison between a period of HIF-PH inhibitor treatment and the natural progression of renal anemia, capturing its potential for improvements over a longer duration to observe cardiac reverse remodeling and the amelioration of heart failure burden.

### 4.2. Feasibility of HIF-PH Inhibitors in Heart Failure Patients

The RED-HF trial previously reported increased thromboembolic risks with ESAs in heart failure patients [9], while the ASCEND trials indicated a potential rise in heart failure incidence during HIF-PH inhibitor therapy [22]. Although the mechanism behind these findings remains unclear, it is suggested that anemia correction may increase vascular resistance and afterload on the left ventricle by raising blood viscosity and diminishing hypoxic vasodilation [23].

However, the current study and recent literature suggest that HIF-PH inhibitors are feasible for heart failure patients, especially with careful patient selection and target hemoglobin levels set between 10 and 12 g/dL (lower than the levels generally recommended for non-dialysis CKD ranging between 11 and 13 g/dL). Importantly, none of the patients in this study had NYHA class IV symptoms or recent thromboembolic events, underscoring the need to identify optimal candidates for this therapy. Further studies are warranted to establish optimal patient selection and target hemoglobin levels for HIF-PH inhibitor therapy.

### 4.3. Impact of HIF-PH Inhibitors on Renal Anemia in Heart Failure Patients

Hemoglobin levels declined during the pre-treatment period, probably due to the natural progression of renal anemia within the cardio-renal-anemia syndrome framework. However, HIF-PH inhibitor therapy reversed this trend, even in the presence of heart failure. Notably, the presence of heart failure is associated with resistance to anti-anemic treatments.

This finding aligns with recent studies that reported improvements in anemia with HIF-PH inhibitors in the heart failure cohorts [16,17]. Anemia in heart failure is multifactorial, with causes including functional iron deficiency, inflammation, gastrointestinal congestion, reduced erythropoietin production, and fluid retention [5]. HIF-PH inhibitors activate HIF signaling, which enhances iron absorption and utilization through the hepcidin–ferroportin axis [24], with recent studies showing decreased hepcidin levels post-therapy in heart failure patients [25].

In the present study, ferritin and total iron binding capacity remained unchanged during HIF-PH inhibitor therapy. Following HIF-PH inhibitor initiation, ferritin levels would decrease by facilitated iron use, whereas enhanced iron absorption, together with the supplementation of iron, may compensate for such a decline. Given the enhanced utilization of stored iron, monitoring of iron metabolism and appropriate iron supplementation should be essential during HIF-PH inhibitor therapy. Further studies are needed to clarify these mechanisms and the precise role of HIF-PH inhibitors, in combination with iron supplementation, in anemia improvement among heart failure patients.

### 4.4. Impact of HIF-PH Inhibitors on Other Clinical Parameters

This study observed that HIF-PH inhibitor therapy resulted in significant improvements in several clinical parameters associated with heart failure, including reductions in plasma B-type natriuretic peptide and C-reactive protein levels, along with an increased estimated glomerular filtration rate. Notably, these improvements occurred without any changes in heart failure medications, suggesting the effectiveness of HIF-PH inhibitors in targeting the interdependent pathophysiology of heart, kidney, and anemia.

HIF-PH inhibitors are thought to correct iron utilization disorders, which is crucial for patients with heart failure and CKD [24]. Stored iron, reflected by serum ferritin levels, is essential for optimal HIF-PH inhibitor response. Previous studies suggested that a ferritin level ≥ 41.8 ng/mL may serve as a threshold for improving heart failure outcomes during HIF-PH therapy [16]; in this study, most participants met this threshold, supporting the relationship between iron availability and HIF-PH inhibitor efficacy.

HIF-PH inhibitors may offer an advantage over ESAs in managing anemia associated with heart failure and CKD. Patients with these conditions typically require higher erythropoietin levels due to chronic inflammation and erythropoietin hypo-responsiveness [26,27]. High erythropoietin levels have been linked to poor clinical outcomes in heart failure [28], and HIF-PH inhibitors may effectively raise hemoglobin with much lower erythropoietin levels by addressing erythropoietin resistance [29]. This mechanism reduces hypoxia-induced peripheral vasodilation, which in turn decreases the activation of the renin-angiotensin system and enhances the efficacy of anti-heart failure medications [5].

The correction of anemia indirectly contributes to the improvement of heart failure and systemic inflammation through the intricate interplay of the cardio-renal anemia syndrome. By addressing anemia, organ oxygenation is enhanced, and cardiac workload is alleviated, which in turn mitigates heart failure-associated systemic inflammation. Furthermore, the reduction in systemic inflammation may stabilize erythropoietin resistance, creating a positive feedback loop that facilitates more effective anemia correction.

Furthermore, HIF-PH inhibitors may exert cardio-protective effects that extend beyond their influence on erythropoietin production and iron regulation. The activation of HIF in ischemic tissues triggers protective cellular responses, including re-oxygenation and repair mechanisms, which may support improved cardiovascular function [30]. This cardio-protective effect may represent an independent pathway by which HIF-PH inhibitors contribute to better outcomes in heart failure patients with concurrent renal anemia.

### 4.5. Future Concerns

Several areas of concern need to be addressed in future research to build upon this study’s findings. First, the long-term safety and efficacy of HIF-PH inhibitors in patients with both heart failure and renal anemia remain uncertain. Since the study duration was only six months, longer follow-up studies are required to determine if the observed benefits are sustained over time and to monitor for potential late-onset adverse effects, including cardiovascular events.

Second, patient selection for HIF-PH inhibitor therapy requires further refinement. It is essential to identify which subgroups of heart failure patients are most likely to benefit from this therapy while minimizing risks, especially given the complex pathophysiology of heart failure, CKD, and anemia. Determining an optimal hemoglobin target and the most effective dosing strategies for these inhibitors will also be crucial to maximizing benefits while avoiding potential adverse effects such as increased blood viscosity and vascular complications.

Moreover, the specific impact of different HIF-PH inhibitors remains uncertain, as this study primarily involved daprodustat and vadadustat. Few studies compared the clinical efficacy of each type of HIF-PH inhibitor, except for the study conducted by Sezai and colleagues [31]. Roxadustat and vadadustat stabilize both HIF-1 alpha and HIF-2 alpha, whereas HIF-2 alpha is more stabilized than HIF-1 alpha following the administration of daprodustat. HIF-1 alpha is associated with an acute response to hypoxia via the activation of glycolysis and angiogenesis. HIF-2 alpha is associated with a chronic response to hypoxia via improving iron metabolism disorder. Drug metabolism and prescription rate are also different between the medications. These differences may have impacts on anemia improvement. Comparative studies between the various HIF-PH inhibitors would clarify whether certain agents are more effective or safer in this patient population.

Another critical concern is the interaction of HIF-PH inhibitors with heart failure medications. Although heart failure therapies remained stable in this study, further exploration of potential pharmacologic interactions and combined effects of these drugs is needed to optimize comprehensive treatment strategies.

Lastly, considering the small sample size and single-center nature of this study, larger, multi-center trials are necessary to validate the results across diverse patient populations. These studies should also include more diverse demographics to ensure that the benefits of HIF-PH inhibitors can be generalized across broader clinical settings.

### 4.6. Limitations

This study has several notable limitations. Firstly, the small sample size limits the generalizability of the findings, as this is a hypothesis-formulating proof-of-concept study. Furthermore, the present study was retrospective. Consequently, prospective larger multi-center studies are needed to confirm the results, as certain statistical comparisons may reach significance in studies with greater statistical power. Additionally, while a multivariable analysis was performed, some confounding factors may not be accounted for, potentially affecting the findings.

Another limitation is the absence of a traditional control arm comprising heart failure patients with renal anemia who did not receive HIF-PH inhibitors. Due to the strong evidence supporting HIF-PH inhibitors for treating renal anemia, it was ethically challenging to withhold treatment [18]. To address this, a self-control design was used, comparing parameters from a pre-treatment period with those from the on-treatment period. We believe that such a study design is the best to evaluate the clinical implication of HIF-PH inhibitors in this real-world cohort in the current era. Furthermore, we identified improvement in multiple clinical parameters during HIF-PH inhibitor therapy. However, this design limits the ability to compare outcomes directly with a non-treated group.

Furthermore, while heart failure medication doses remained stable between pre-treatment and on-treatment periods, the long-term effects of these medications may have influenced the results. Finally, we did not have any established allocation protocol to prescribe which types of HIF-PH inhibitors to use. The study predominantly involved patients treated with daprodustat and vadadustat, and the potential differences in therapeutic effects between various HIF-PH inhibitors remain unclear, necessitating further research to elucidate any distinctions among the available HIF-PH inhibitors.

## 5. Conclusions

This study demonstrates that HIF-PH inhibitors are effective in significantly increasing hemoglobin levels in patients with renal anemia and heart failure, accompanied by improvements in heart failure burden, renal function, and systemic inflammation status. These findings suggest that HIF-PH inhibitors may not only address anemia but also positively impact cardio-renal health by breaking the cycle between heart failure, CKD, and anemia. Importantly, no drug-related adverse events were observed, and patients tolerated the therapy well, indicating the feasibility of HIF-PH inhibitors for this patient population. These findings highlight the potential of the broader clinical utility of HIF-PH inhibitors for targeting multiple therapeutic targets, such as anemia, heart failure, and CKD.

The study highlights that HIF-PH inhibitors may offer a valuable alternative to ESAs by increasing hemoglobin with lower circulating erythropoietin levels, potentially lowering the risk of adverse cardiovascular outcomes. However, due to the small sample size, lack of a placebo-controlled group, and short duration of follow-up, these findings require validation through larger, multi-center studies. Future research should also explore optimal patient selection criteria, dosage, and hemoglobin targets to maximize therapeutic benefits while minimizing risk.

## Figures and Tables

**Table 1:** Baseline characteristics.

	*N* = 69
Demographics	
Age, years	82 (78–85)
Men sex	27 (39%)
Body mass index, kg/m^2^	22.8 (19.9–24.2)
Vital sign	
Systolic blood pressure, mmHg	119 (93–125)
Pulse rate, rpm	69 (65–83)
Comorbidity	
Heart failure	69 (100%)
Diabetes mellitus	17 (25%)
Atrial fibrillation	25 (36%)
History of stroke	14 (20%)
Coronary artery disease	21 (30%)
Laboratory data	
Hemoglobin, g/dL	9.2 (8.4–10.5)
Serum albumin, g/dL	3.7 (3.2–3.8)
eGFR, mL/min/1.73 m^2^	29.1 (19.0–35.1)
Plasma B-type natriuretic peptide, pg/mL	264 (156–372)
Serum C-reactive protein, mg/dL	0.31 (0.03–1.20)
Serum iron, μg/dL	45.5 (27.0–72.0)
Ferritin, ng/mL	59.5 (44.0–137.0)
Total iron binding capacity, μg/dL	283 (207–318)
Transferrin saturation, %	25.0 (17.1–31.6)
Thyroid stimulating hormone, μIU/mL	2.5 (1.3–3.4)
Free T3, pg/mL	2.1 (1.8–2.7)
Free T4, ng/mL	1.1 (0.8–1.3)
Echocardiography data (*N* = 49)	
Left ventricular end-diastolic diameter, mm	49 (41–59)
Left ventricular ejection fraction, %	60 (51–64)
E/e’ ratio	12.6 (7.5–22.3)
TRPG, mmHg	22 (20–32)
Moderate or greater mitral regurgitation	13 (27%)
Moderate or greater tricuspid regurgitation	12 (25%)
Left ventricular ejection fraction <40%	11 (22%)
Medication	
Beta-blocker	53 (77%)
Renin-angiotensin system inhibitor	63 (91%)
Mineralocorticoid receptor antagonist	34 (49%)
SGLT2 inhibitor	18 (26%)
Furosemide	50 (73%)
Furosemide equivalent dose, mg/day	20 (0, 20)
Tolvaptan	37 (54%)
Tolvaptan dose, mg/dL	3.75 (0, 7.5)
Iron supplementation	53 (77%)

Baseline characteristics data were obtained at the time when HIF-PH inhibitors were initiated. Continuous variables were stated as median and interquartile and categorical variables were stated as numbers and percentages. eGFR—estimated glomerular filtration rate; TRPG—tricuspid regurgitant pressure gradient; SGLT2—sodium-glucose cotransporter 2.

**Table 2:** Trajectory of laboratory data.

	Before 6 Months	Baseline	*p*-Value vs. Before 6 Months	After 6 Months	*p*-Value vs. Baseline
Serum albumin, g/dL	3.6 (3.1–3.8)	3.7 (3.2–3.8)	-	3.7 (3.5–4.0)	-
eGFR, mL/min/1.73 m^2^	35.5 (25.6–41.5)	29.1 (19.0–35.1)	<0.001 *	34.6 (28.6–38.7)	<0.001 *
Plasma BNP, pg/mL	238 (163–305)	264 (156–372)	0.43	172 (105–350)	0.018 *
Serum C-reactive protein, mg/dL	0.14 (0.03–0.72)	0.31 (0.03–1.20)	0.82	0.05 (0.04–0.06)	<0.001 *
Serum iron, μg/dL	56.0 (45.5–85.5)	45.5 (27.0–72.0)	-	79.0 (75.0–81.5)	-
Ferritin, ng/mL	56.0 (45.5–85.5)	59.5 (42.0–137.0)	-	76.0 (71.0–86.5)	-
Total iron binding capacity, μg/dL	269 (224–322)	283 (207–318)	-	280 (255–325)	-
Thyroid stimulating hormone, μIU/mL	2.6 (1.4–3.7)	2.5 (1.3–3.4)	-	2.4 (1.4–3.7)	-
Free T3, pg/mL	2.0 (1.7–2.9)	2.1 (1.8–2.7)	-	2.0 (1.8–2.9)	-
Free T4, ng/mL	1.0 (0.7–1.4)	1.1 (0.8–1.3)	-	1.0 (0.9–1.2)	-

Laboratory data were obtained at three time points: 6 months before the initiation of HIF-PH inhibitors, the time when HIF-PH inhibitors were initiated (baseline), and 6 months after the initiation of HIF-PH inhibitors (after 6-month HIF-PH inhibitor treatment). Continuous variables were stated as median and interquartile. eGFR—estimated glomerular filtration rate; BNP—B-type natriuretic peptide. When the trend analyses using Friedman test reached statistical significance, post hoc Wilcoxon signed-rank tests were performed for two-group comparison. Otherwise, *p*-values were not stated. * *p* < 0.05.

**Table 3:** Trajectory of echocardiography data.

	Before 6 Months (*N* = 48)	Baseline (*N* = 49)	*p*-Value vs. Before 6 Months	After 6 Months (*N* = 62)	*p*-Value vs. Baseline
LVDD, mm	48 (44–52)	49 (41–59)	-	48 (45–49)	-
LVEF, %	64 (50–72)	60 (51–64)	-	62 (59–67)	-
E/e’ ratio	12.7 (10.5–18.7)	12.6 (7.5–22.3)	-	13.6 (7.7–20.1)	-
TRPG, mmHg	27 (20–38)	22 (20–32)	-	25 (20–32)	-
Moderate or greater MR	16 (33%)	13 (27%)	-	15 (24%)	-
Moderate or greater TR	5 (10%)	12 (25%)	0.021 *	4 (7%)	0.008 *
LVEF <b 40%	9 (19%)	11 (22%)	-	12 (19%)	-

Echocardiography data were obtained at three time points: 6 months before the initiation of HIF-PH inhibitors, the time when HIF-PH inhibitors were initiated (baseline), and 6 months after the initiation of HIF-PH inhibitors (after 6-month HIF-PH inhibitor treatment). Continuous variables were stated as median and interquartile and categorical variables were stated as numbers and percentages. LVDD—left ventricular end-diastolic diameter; LVEF—left ventricular ejection fraction; TRPG—tricuspid regurgitant pressure gradient; MR—mitral regurgitation; TR—tricuspid regurgitation. For continuous variables, when the trend analyses using Friedman test reached statistical significance, post hoc Wilcoxon signed-rank tests were performed for two-group comparison. For categorical variables, when the trend analyses using Cochran Q test reached statistical significance, post hoc McNemar test was performed for two-group comparison. Otherwise, *p*-values were not stated. * *p* < 0.05.

**Table 4:** Trajectory of medication data.

	Before 6 Months	Baseline	*p*-Value vs. Before 6 Months	After 6 Months	*p*-Value vs. Baseline
Beta-blocker	51 (74%)	53 (77%)	-	53 (77%)	-
Renin-angiotensin system inhibitor	62 (90%)	63 (91%)	-	63 (91%)	-
Mineralocorticoid receptor antagonist	32 (46%)	34 (49%)	-	33 (48%)	-
SGLT2 inhibitor	18 (26%)	18 (26%)	-	23 (33%)	-
Furosemide	48 (70%)	50 (73%)	-	51 (74%)	-
Furosemide equivalent dose, mg/day	20 (20, 40)	20 (0, 20)	-	20 (0, 40)	-
Tolvaptan	26 (38%)	37 (54%)	-	35 (51%)	-
Tolvaptan dose, mg/dL	0 (0–3.75)	3.75 (0–7.5)	0.074	0 (0–3.75)	0.12
Iron supplementation	50 (72%)	53 (77%)	-	56 (81%)	-

Prescription data were obtained at three time points: 6 months before the initiation of HIF-PH inhibitors, the time when HIF-PH inhibitors were initiated (baseline), and 6 months after the initiation of HIF-PH inhibitors (after 6-month HIF-PH inhibitor treatment). Continuous variables were stated as median and interquartile and categorical variables were stated as numbers and percentages. SGLT2—sodium-glucose cotransporter 2. For continuous variables, when the trend analyses using Friedman test reached statistical significance, post hoc Wilcoxon signed-rank tests were performed for two-group comparison. For categorical variables, when the trend analyses using Cochran Q test reached statistical significance, post hoc McNemar test was performed for two-group comparison. Otherwise, *p*-values were not stated.

**Table 5:** Potential variables associated with the degree of increase in hemoglobin levels during HIF-PH inhibitor therapy.

	Univariable Analyses		Multivariable Analyses	
	Beta-Value	*p*-Value	Beta-Value	*p*-Value
Age, years	0.012	0.5		
Diabetes mellitus	−0.353	0.5		
Hemoglobin, g/dL	−0.891	<0.001 *	−0.947	<0.001 *
Serum albumin, g/dL	−0.942	0.008 *	−0.056	0.89
eGFR, mL/min/1.73 m^2^	−0.181	0.35		
Plasma BNP, pg/mL	0.001	0.52		
Serum C−reactive protein, mg/dL	0.211	0.15		
Ferritin, ng/mL	−0.002	0.38		
LVEF, %	0.008	0.6		
E/e’ ratio	−0.108	0.045 *	−0.132	0.002 *

Linear regression analysis for the hemoglobin change during 6-month HIF-PH inhibitor therapy was performed among potential baseline variables. Variables significant with *p* < 0.05 in the univariable analyses were included in the multivariable analysis with a forced method. eGFR—estimated glomerular filtration rate; BNP—B-type natriuretic peptide; LVEF—left ventricular ejection fraction. * *p* < 0.05.

## Data Availability

Data are available from the corresponding author upon reasonable request.
